# Holistic Approach to Investigate Pyrrolizidine Alkaloids in Honeys from Diverse Botanical Origin: From Target to Suspect and Nontarget Screening Analysis

**DOI:** 10.1021/acs.jafc.5c05585

**Published:** 2025-06-30

**Authors:** Laura Carbonell-Rozas, José Raúl Belmonte-Sánchez, Paula Soto-Rosiña, Roberto Romero-González, Antonia Garrido Frenich

**Affiliations:** Research Group “Analytical Chemistry of Contaminants”, Department of Chemistry and Physics, Research Centre for Mediterranean Intensive Agrosystems and Agrifood Biotechnology (CIAIMBITAL), Agrifood Campus of International Excellence (ceiA3), 16721 University of Almeria, E-04120 Almeria, Spain

**Keywords:** pyrrolizidine alkaloids, honey, plant toxins, screening analysis, molecular networking

## Abstract

This study investigates the presence of pyrrolizidine alkaloids (PAs) in honey from diverse botanical origins using a combination of target analysis, through ultrahigh-performance liquid chromatography coupled with tandem mass spectrometry and suspect and nontarget screening via ultrahigh-performance liquid chromatography coupled with quadrupole-Orbitrap high-resolution mass spectrometry. A total of 80 honey samples were analyzed, with 61% were found to contain at least one PA. Eucalyptus and wildflower honeys showed the highest contamination levels, reaching 27 μg/kg when expressed as the sum of regulated PAs. Indicine and lycopsamine, commonly associated with plant species, were the most frequently detected compounds. Statistical analysis revealed variability in PA profiles across different honey types, though classification based on botanical origin remained limited. Additional strategies, including molecular networking and the computationally generated spectral library, expanded the detection capabilities of these compounds. In particular, molecular networking can be a powerful tool to reveal structural relationships among PAs.

## Graphical abstract

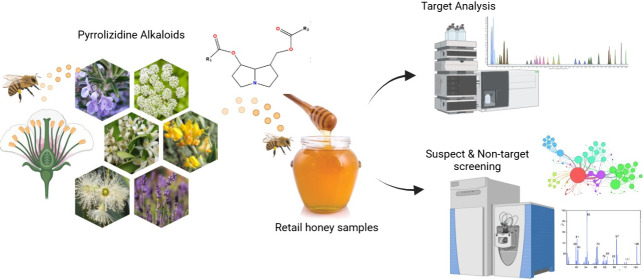

## 1 Introduction

In recent years, there has been a growing concern about the occurrence of pyrrolizidine alkaloids (PAs) in food samples, as the number of food alerts reported in the EU has significantly increased, particularly from 2020 to 2023.^1^ PAs are secondary metabolites produced by plants in response to stress factors, such as climatic changes and attacks by herbivores, insects, and pathogens, serving as a self-defense mechanism.^2^ PAs are widely distributed in the plant kingdom throughout the world. It is estimated that approximately 6000 plant species, representing 3% of all flowering plants, produce these natural toxins. Notably, PAs are commonly found in species of the families Asteraceae, Boraginaceae, Heliotropiaceae, and Apocynaceae as well as certain genera within the Orchidaceae and Fabaceae families.^3^ It is also influenced by other factors, such as climate and soil properties, nutrients, water quantity, and herbivore infestations.^4^

PAs are mostly composed of a pyrrolizidine ring or tertiary base (necine) and an esterified organic acid (necic acid), which are responsible for their structural diversity. To date, more than 600 different PAs have been identified. They can be classified into saturated and unsaturated types according to the presence or absence of double bonds at positions C-1 and C-2 of the necine structure, with the unsaturated types being more toxic.^5,6^ They are usually found in the form of tertiary bases or pyrrolizidine alkaloids *N*-oxides (PANOs), which coexist in most plants.^6^

Although these alkaloids exhibit an interesting spectrum of biological properties that can be used in pharmacology,^7^ their toxicity to humans is well documented, including hepatotoxicity, genotoxicity, carcinogenicity, and cytotoxicity.^3,5,8^ Humans are exposed to PAs through food intake, either by consuming PA-containing plants or food contaminated with PAs (e.g., milk, meat, and honey), which raises significant concerns in food safety nowadays.^9,10^

In this respect, in 2017, the European Food Safety Agency (EFSA) published a statement on the risks to human health related to the presence of PAs in honey, tea, plant infusions, and food supplements.^11^ Recently, the European Commission Regulation (EU) 2023/915, repealing Regulation (EC) No. 2020/2040, sets new maximum levels for PAs across various food categories, including tea, herbal infusions, species, and pollen-based products.^12^ These limits refer to the lower bound sum of 35 PAs/PANOs, selected due to their concern for toxicity and frequent occurrence in food. For pollen and its derived products placed on the market, the maximum allowable level has been set at 500 μg/kg; however, no regulations have yet been established for honey.

EFSA also emphasized the need for more comprehensive data on the occurrence of PAs in these foods and highlighted the need for the development of more sensitive and selective analytical methods to improve the detection of PAs. The growing interest in evaluating the presence of PAs/PANOs in food has driven significant advances in analytical methodologies to detect and quantify these contaminants in different food matrices.^13–16^ In this sense, liquid chromatography coupled to tandem mass spectrometry detection (LC–MS/MS) is considered the gold technique for PA determination.^17^ However, given the large number of known PAs, the main challenge still lies in effectively separating and detecting them individually. In most cases, solid–liquid extraction (SLE) or liquid–liquid extraction (LLE) usually combined with solid-phase extraction (SPE) as the purification step has been widely used due to the complexity of food matrices. Although PAs have been primarily investigated in plant-based foods, animal-derived products, such as milk, eggs, meat and beehive products, should also be investigated due to the potential transfer of PAs to these products,^18,19^ in particular, to beehive products such as pollen and honey.^20,21^ In fact, the contamination profile of honey is influenced by the botanical origins of the plants that bees forage on, which can vary significantly depending on the geographical region. Therefore, it is crucial to expand the monitoring of PAs in honey and other beehive products to better assess their risks.

In this regard, considering the growing widespread occurrence of PAs, we propose a comprehensive evaluation of PAs in retail honey samples from different botanical origin. To achieve this, a target approach based on a UHPLC–MS/MS method to analyze 35 regulated PAs was combined with the suspect and nontarget screening via high resolution mass spectrometry using UHPLC–Q-Orbitrap. This expanded the results of target analysis into a more holistic evaluation. *In silico* spectra based on available data, together with well-known libraries, could improve the assessment of both known and potentially novel PA compounds. In addition, the use of molecular networking is introduced to provide a more complete understanding of their prevalence and diversity within the honey matrix, offering a more thorough and complete assessment.

## 2 Materials and Methods

### 2.1 Chemicals and Reagents

Methanol (MeOH) and acetonitrile (MeCN) of LC–MS grade were sourced from Honeywell Riedel-de Haën (Seelze, Germany) and Carlo Erba Reagents (Barcelona, Spain), respectively, while ultrapure Milli-Q water was obtained from Panreac AppliChem (Barcelona, Spain). Formic acid (99%) was supplied by Fisher Scientific (Erembodegem, Belgium), and ammonium formate (97%) and ammonium hydroxide NH_4_OH, 99.99%) were supplied by Sigma-Aldrich (St. Louis, MO, U.S.A.). Ethyl acetate (EtAc, 99.5%) and triethylamine (TEA, 99%) were sourced from Honeywell Riedel-de Haën (Seelze, Germany), while sulfuric acid (H_2_SO_4_, (95%) from J.T. Baker (Deventer, Netherlands) was used.

The PA standards were obtained from PhytoPan (Heidelberg, Germany). A total of 35 PAs were considered in this study: intermedine, intermedine *N*-oxide, rinderine, indicine, lycopsamine, europine, europine *N*-oxide, indicine *N*-oxide, echimidine *N*-oxide, lycopsamine *N*-oxide, spartiodine, spartiodine *N*-oxide, retrorsine, retrorsine *N*-oxide, heliotrine, seneciphylline, heliotrine *N*-oxide, seneciphylline *N*-oxide, integerrimine, senecionine, senecionine *N*-oxide, senkirkine, echimidine, lasiocarpine, lasiocarpine *N*-oxide, usaramine, usaramine *N*-oxide, heliosupine, heliosupine *N*-oxide, integerrimine *N*-oxide, senecivernine, senecivernine *N*-oxide, senecivernine, senecivernine *N*-oxide, and rinderine *N*-oxide. Each analyte was prepared at a concentration of 2500 μg/mL by dissolving 10 mg of each compound in 4 mL of MeOH. Additionally, a 15 μg/mL mixture containing the 35 PAs/PANOs was prepared using a solvent mixture of water/MeOH (95:5, v/v). Different working solutions were prepared by diluting the intermediate stock mix with water/MeOH (95:5, v/v) at the required concentrations. All solutions were stored at −20 °C in sealed dark glass vials.

### 2.2 Honey Samples

A total of 80 honey samples of various botanical origins (both monofloral and multifloral) were included in this study. Wildflower honey was classified as multifloral. The samples comprised common honey types such as orange blossom (; *n* = 15), eucalyptus (*n* = 11), thyme (; *n* = 7), rosemary (; *n* = 17) and wildflower honey (*n* = 18), as well as less common varieties such as lavender ( spp.; *n* = 5), heather (; *n* = 3), albaida (; *n* = 2), oak (; *n* = 1), and anise (; *n* = 1). The samples were purchased from various suppliers in Spain. According to the labeling, most of the honey samples were of Spanish origin, while some contained honey from both European and non-European sources, including Uruguay, Argentina, Turkey, Mexico, and Vietnam. These samples represent a diverse range of honey varieties that are commercially available in Spain. The samples were stored in their original packaging in a dry and fresh place until analysis.

### 2.3 Sample Extraction Procedure

The sample preparation method for honey samples was based on a previous work.^22^ Some modifications were carried out to reduce the sample size and organic solvent consumption. Thus, 1 g of homogenized honey sample was weighed in a 15 mL centrifuge tube with 10 mL of 50 mM sulfuric acid. The mixture was shaken for 30 s until completely dissolved, followed by ultrasonic bath extraction for 10 min at 35 °C. The tube was centrifuged for 10 min at 5000*g*, and 2 mL of supernatant were loaded onto an Oasis MCX cartridge (6 cm^3^, 150 mg) from Waters Corporation (Wilmslow, U.K.) previously conditioned with 3 mL of MeOH and equilibrated with 3 mL of water. Afterward, it was washed with 3 mL of water and 3 mL of MeOH. The PAs were eluted with 3 mL of a solvent mixture consisting of EtOAc/MeOH/MeCN (80:10:10, v/v/v) with 1% NH_4_OH and 1% triethylamine in a glass tube (maximum flow rate was 1 drop/s at all stages). The purified extract was then evaporated to dryness under a gentle nitrogen stream, and reconstituted with 1 mL of a mixture of H_2_O/MeOH (95:5, v/v). The extract was filtered through a glass fiber syringe filter (0.7 μm pore size × 13 mm) from Whatman Puradisc (Marlborough, MA, United States) into a LC vial. The schematic procedure for PAs extraction and purification in honey sample analysis is shown in Figure 1.

**Figure 1:**
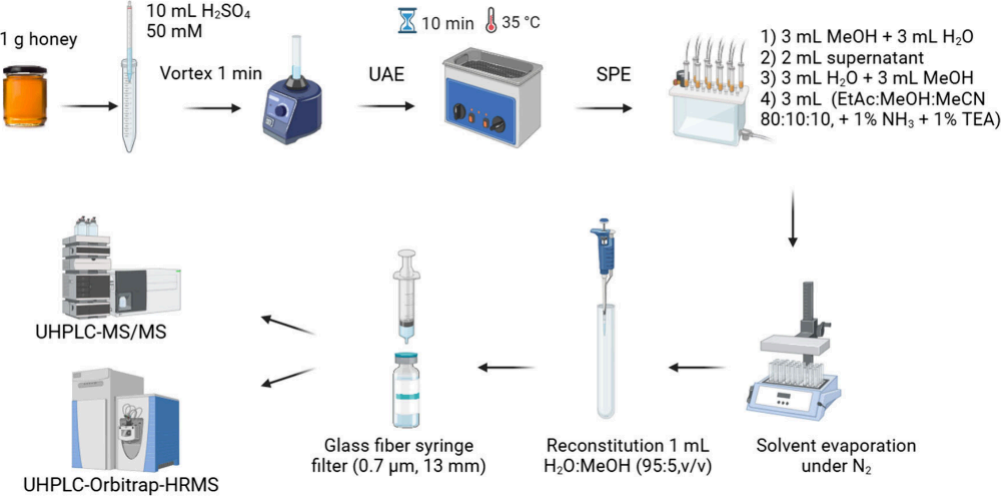
Schematic overview of the sample treatment for PAs isolation from honey samples.

An analytical AB204-S balance (Mettler Toledo, Greifensee, Switzerland), a vortex mixer WX (Velp Scientifica, Usmate, Italy), an Elmasonic S 80H ultrasonic bath (Elma Schmidbauer, Germany), and a Frontier 5718R centrifuge (Ohaus, Nänikon, Switzerland) were used during the procedure.

### 2.4 Target Analysis by UHPLC–QqQ-MS/MS

Target analyses were carried out using a 1290 RRLC liquid chromatograph instrument (Agilent, Santa Clara, CA, U.S.A.) with a Jet Stream electrospray ionization (ESI) source (G1958-65138) and interfaced to an Agilent triple quadrupole (QqQ) mass spectrometer (6460 A). Chromatographic separation was performed in an Acquity BEH C8 column (100 × 2.1 mm, 1.7 μm particle size) from Waters Corporation (Waters, Wilmslow, U.K.). The mobile phase was composed of solvent A (5 mM ammonium formate aqueous solution and 0.1% formic acid) and solvent B (0.1% formic acid in MeCN) with a flow rate of 0.3 mL/min, an injection volume of 5 μL, and a column temperature of 30 °C. The elution gradient was as follows: 0–1 min, 5% B; 1–15 min, 5–10% B; 15–18 min, 10–30% B; 18–18.5 min, 30–95%; and 18.5–20, 95% B. Subsequently, eluent B was restored to 5% in 1 min and held for 2.5 min. The total run analysis was 23.5 min.

The mass spectrometer (MS) operated in positive electrospray ionization (ESI+) mode. All PAs were analyzed under multiple reaction monitoring (MRM) conditions, selecting three product ions for each one (Table S1). The ionization mode and the best transition signals for each target compound were studied by direct infusion of the corresponding standard. Individual standard solutions of 1 μg/mL were infused into the ion source at a flow rate of 5 μL/min, in combination with an isocratic mobile phase (1:1 MeCN/H_2_O) at a flow rate of 0.3 mL/min. The MS conditions were as follows: gas flow, 5 L/min; source temperature, 300 °C; sheath gas temperature, 350 °C; nebulizer, 45 psi; sheath gas flow, 11 L/min; capillary, 3500 V; and nozzle voltage, 500 V. The dwell time for all MRM transitions was 0.10 s. Nitrogen was used as a nebulizing and collision gas.

The UHPLC–QqQ-MS/MS data were acquired using the Agilent Mass Hunter Workstation and processed with the Agilent Mass Hunter Quantitative. The Optimizer (version 10.1) of Agilent Mass Hunter was also used for the automatic optimization of the MRM parameters.

### 2.5 Suspect and Nontarget Screening Analysis by UHPLC–Q-Orbitrap-HRMS

The analysis was conducted in a Vanquish LC chromatograph (Thermo Fisher Scientific, Waltham, MA, U.S.A.) comprising a binary pump, a degasser, a temperature-controlled column compartment, and an autosampler. This system was coupled to a Q-Exactive hybrid quadrupole-Orbitrap high-resolution mass spectrometer (Thermo Fisher Scientific). Chromatographic separation was performed using the same column and mobile phases indicated in section 2.4, following the previously specified separation parameters.

The HRMS operated in both positive and negative ESI modes, considering the following parameters: heater temperature was 300 °C and capillary temperature, 300 °C. The auxiliary and sheath gas used was nitrogen (95%), the spray voltage was 4 kV, and the S-lens radio frequency level was 50 (arbitrary units).

Full-scan mode was used for HRMS data acquisition of precursor ions in the mass-to-charge ratio (*m*/*z*) range of 50–750 for both ESI+ and ESI– modes, with a maximum injection time (IT) of 250 ms, an automatic gain control (AGC) target of 1 × 10^5^, and a resolution of 70 000 fwhm (*m*/*z* 200).

Data-dependent acquisition (DDA) was used to monitor fragment ions (MS/MS data) and was performed using the data-dependent dd-MS^2^ (Top5) mode within the *m*/*z* 200–2000 range in both ESI+ and ESI– modes. This mode involved the higher energy collisional dissociation (HCD) collision cell with normalized collision energies (NCEs) of 30 eV. MS/MS data were acquired with a maximum IT of 125 ms, an isolation window of 5 *m*/*z*, an AGC target of 1 × 10^5^, a dynamic exclusion time of 10 s, and a resolution of 35 000 fwhm at *m*/*z* 200 for both polarities.

An inclusion list of predefined target precursor ions was created for the 35 PAs with available standards, ensuring their selective fragmentation and subsequent identification in complex samples.

UHPLC–Q-Orbitrap-HRMS data was acquired using the Xcalibur Sequence Setup software (Version 4.4, Thermo Fisher Scientific).

### 2.6 HRMS Workflow for Data Processing and Elucidation

A comprehensive workflow for the suspect and nontarget screening analysis of PAs in honey samples was developed using Compound Discoverer version 3.3 software (Thermo Fisher Scientific).

Initially, honey data (.raw files) were imported into the software. The calibration curve containing the 35 PAs was categorized as “standard” for the validation of the workflow and the semiquantification of the annotated PAs. Procedure blanks and quality control (QC) samples were included in the workflow for background signal removal and area refinement. Procedure blanks were categorized as “blank” and QC samples as “quality control”. Background filtering was carried out by setting the maximum allowed ratio of sample to blank at 5 for consideration as a blank feature.

The selected settings for Compound Discoverer were as follows: mass tolerance 5 ppm, minimum peak intensity of 100 000, intensity tolerance of 30%, signal-to-noise (S/N) threshold of 3, intensity threshold of 0.1%, and retention time (RT) tolerance of 0.5 min. The preferred adducts included [M + H]^+^, [M + H – H_2_O]^+^, [M + Na]^+^, [M – H]^−^, [M + K]^+^, and [M + NH_4_]^+^.

Compound identification was performed by matching to LC–MS spectral libraries, including ChemSpider, ChEBI, ChemBank, DrugBank, EPA Toxcast, FDA, MassBank, NIST, PubMed, Toxin (Toxin-Target Database), LIPID maps, PlantCyc, PubMed, Natural Products Atlas 2021_8, mzCloud, and mzVault, with a mass tolerance of 5 ppm for precise annotations.

MzVault 2.3 SP1 was used for *in silico* spectra generation of those PAs without available standards, including the fragmentation patter of 148 PAs and the subsequent in-house database (see section 3.2.1). The option “Factor of Ionization Score” (FISh), available in Compound Discoverer, was used to evaluate the fragmentation coverage of the tentatively identified PAs. The score is based on the match between experimental fragment ions and expected fragmentation patterns, providing an indicator of confidence in the identification.

Molecular networking was generated using the candidates identified after data refinement, using the Molecular Networking option in Compound Discoverer. The optimization of the settings is described in section 3.2.2.

### 2.7 Statistical Data Analysis

The treatment of the data set was carried out using the open source Orange Data Mining software (https://orangedatamining.com).^23^ The data set, provided in CSV format, included the samples, the detected and quantified analytes, and the categorical classification of the types of honey. The honey types with fewer than five samples, specifically heather, albaida, oak, and anise, were excluded from the statistical analysis.

Exploratory analysis plots, such as unsupervised hierarchical clustering analysis (HCA) (Euclidean distance and Ward’s linkage method), were generated in Orange Data Mining to visually explore potential trends and patterns in the data set, particularly in the differentiation of honey types. These analyses relied on unsupervised approaches to better understand the inherent structure of the data.

Statistical significance testing, including ANOVA analysis of variables against honey type, as well as correlation analysis (correlation plot) between variables, was performed in Python within a Google Colab environment to quantitatively assess the relationships between analytes and honey categories and to identify significant associations within the data set.

## 3 Results and Discussion

### 3.1 Target Analysis by UHPLC–MS/MS

The most intense precursor ion, the product ion MRM transitions, as well as optimal fragmentor voltages, and collision energies were obtained automatically by using the optimization tool Optimizer (version 10.1) in Agilent Mass Hunter software. Protonated adducts [M + H]^+^ were monitored as precursor ions for all PAs and three fragment ions were monitored for all compounds (see Table S1). A BEH C8 column with mobile phase consisting of MeCN and 5 mM ammonium formate, both containing 0.1% FA (v/v), was selected, as it has been reported to achieve the separation of 31 out of 35 regulated PAs in a single run.^24^ In this study, gradient refinement enabled the separation of 32 out of the 35 PAs, including the challenging isomer pair intergerrimine *N*-oxide and senecivernine *N*-oxide, which was not previously separated (Figure S1). Nevertheless, baseline separation of rinderine and echinatine, indicine and lycopsamine, as well as indicine *N*-oxide and intermedine *N*-oxide, was still not achievable. However, since the maximum limits for PAs are defined based on the sum of the 35 individual PAs,^12^ the partial overlap of isomeric peaks (e.g., rinderine and echinatine) does not significantly impact the overall quantification.

In any case, this method represents the most comprehensive chromatographic PAs separation in a single run to date, offering an alternative to official methods available for regulated PA determination, which require both alkaline and acidic separations to simultaneously determine 21 and 14 PAs, respectively.^25,26^ In addition, Rizzo et al.^27^ reported that the 14 co-eluting isomers significantly contributed to the total PA content of honey (36%), so that their separation is essential to assess the PA profile in honey.

#### 3.1.1 Validation of the UHPLC–MS/MS Method

The proposed method was validated according to the parameters determined by the Commission Implementing Regulation (EU) 2023/2782. Linearity, matrix effect (ME), recoveries, precision (i.e., repeatability and intermediate precision), and limits of quantification (LOQs), were evaluated.

Matrix-matched calibration curves were conducted at different concentration levels ranging from 0.1 to 100 μg/kg, using wildflower honey as a representative sample, because it encompasses a mix of pollen from diverse botanical sources, making it broadly reflective of various honey types. The peak area was selected as the analytical response and considered as a function of the analyte concentration on the sample. The LOQ for each PA was determined at the lowest concentration of the corresponding calibration curve, achieving a signal-to-noise (S/N) ratio of ten times for the quantification transition. Consequently, a value of 0.1 μg/kg was established for all target compounds. Satisfactory linearity, defined as *R* ^2^, was achieved in all cases (*R* ^2^ > 0.99). The high sensitivity of the method ensures the detection and quantification of the regulated PAs well below the maximum limits established for them in other food samples.

The ME, expressed in %, was determined by comparing the calibration curves with those obtained in the solvent through the eq 1. A ME of 0% indicates the absence of this effect, a ME below 0% involves signal suppression, while a ME above 0% reveals signal enhancement from interferences. ME values were below |14%|, showcasing that the SPE-proposed cleaning was effective considering the complexity of the honey matrix (Table S2).

1 $$\mathrm{ME}\left(\%\right)=\left(\frac{\mathrm{slope in matrix}}{\mathrm{slope in solvent}}-1\right)\times 100$$

Recovery and precision were evaluated at three concentration levels within the linear range: low, middle and high (1, 10, and 100 μg/kg, respectively). For recovery evaluation, wildflower honey samples (*n* = 5) were fortified and treated following the sample treatment procedure and analyzed using UHPLC–MS/MS. These samples were compared, in terms of peak area, with the extracts of blank samples submitted to the sample treatment and spiked at the same concentration levels just before injection. Recoveries, expressed as percentage, ranged from 75 to 109% except for the case of echinatine *N*-oxide at 1 μg/kg (57%) (Table S2). Precision, expressed as relative standard deviation (RSD, %), was evaluated as repeatability (intraday; *n* = 5) and intermediate precision (interday; *n* = 9) providing acceptable values, below 20% in all cases (Table S3). Therefore, the recovery obtained is considered acceptable and does not compromise the reliability of the quantification according to current legislation.^28^

#### 3.1.2 Occurrence Study of Honey Samples

The criteria for the positive identification of PAs in the samples were as follows: a peak must have a signal-to-noise (S/N) ratio of at least 3 for the detection ions, and the relative ion intensities for both detection and quantification ions should match those of the standard solutions. Samples that met these criteria and exceeded the respective limits of quantification (LOQs) were considered positive. The quantification of PAs in the samples was carried out using matrix-matched calibration curves.

Eighty honey samples, specified in section 2.2, were processed in duplicate and analyzed following the proposed SPE–UHPLC–MS/MS method. The concentration of each PA detected and quantified in every sample is specified in Supporting Information (Table S4), as well as those PAs that were detected but not quantified (<LOQ). 61% of the samples were positive with at least one PA. The distribution of PAs identified in the samples is represented in Figure 2. Indicine + lycopsamine were the most frequently detected PAs (appearing in a 30% of the analyzed samples), followed by echimidine (28%). These results agree with previously reported studies that also found lycopsamine and echimidine to be the most prevalent PAs in honey samples.^29,30^ These compounds present retronecine as the necine base and a monoester as necic acid. Thus, it can be stated that type IV PAs (monoesters), also referred to as the lycopsamine type due to their chemical structure, are the most prevalent PAs found in honey samples.

**Figure 2:**
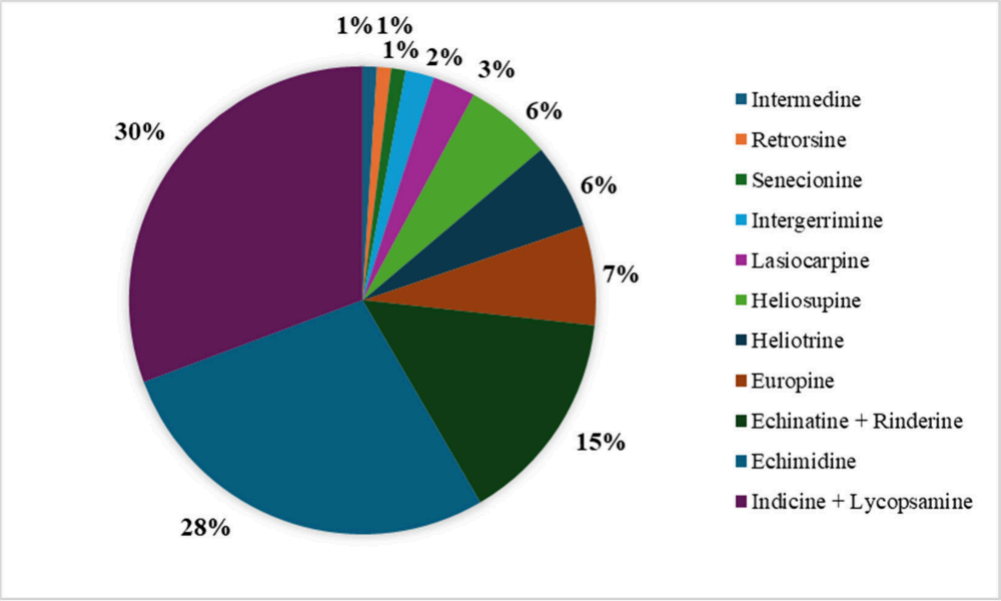
Distribution of PAs in positive honey samples. Ring chart showing the relative distribution (%) of individual PAs detected in positive honey samples. Compounds that were not baseline-separated (e.g., echinatine + rinderine and indicine + lycopsamine) are reported together.

The distribution of total positive samples by honey type is visualized in Figure 3, indicating that eucalyptus and wildflower honey accounted for the highest proportions of positive samples, with 91 and 78% of the samples analyzed being positive, respectively. Eucalyptus samples exhibited the highest total positive counts for PAs, suggesting a strong association with the presence of these alkaloids. On the contrary, other honey types, such as rosemary (35%) and orange blossom (53%), showed significantly lower levels of positive detections, underscoring the variability in PA occurrence across different botanical origins. These findings emphasize the importance of considering honey type when assessing the risk of PA contamination.

**Figure 3:**
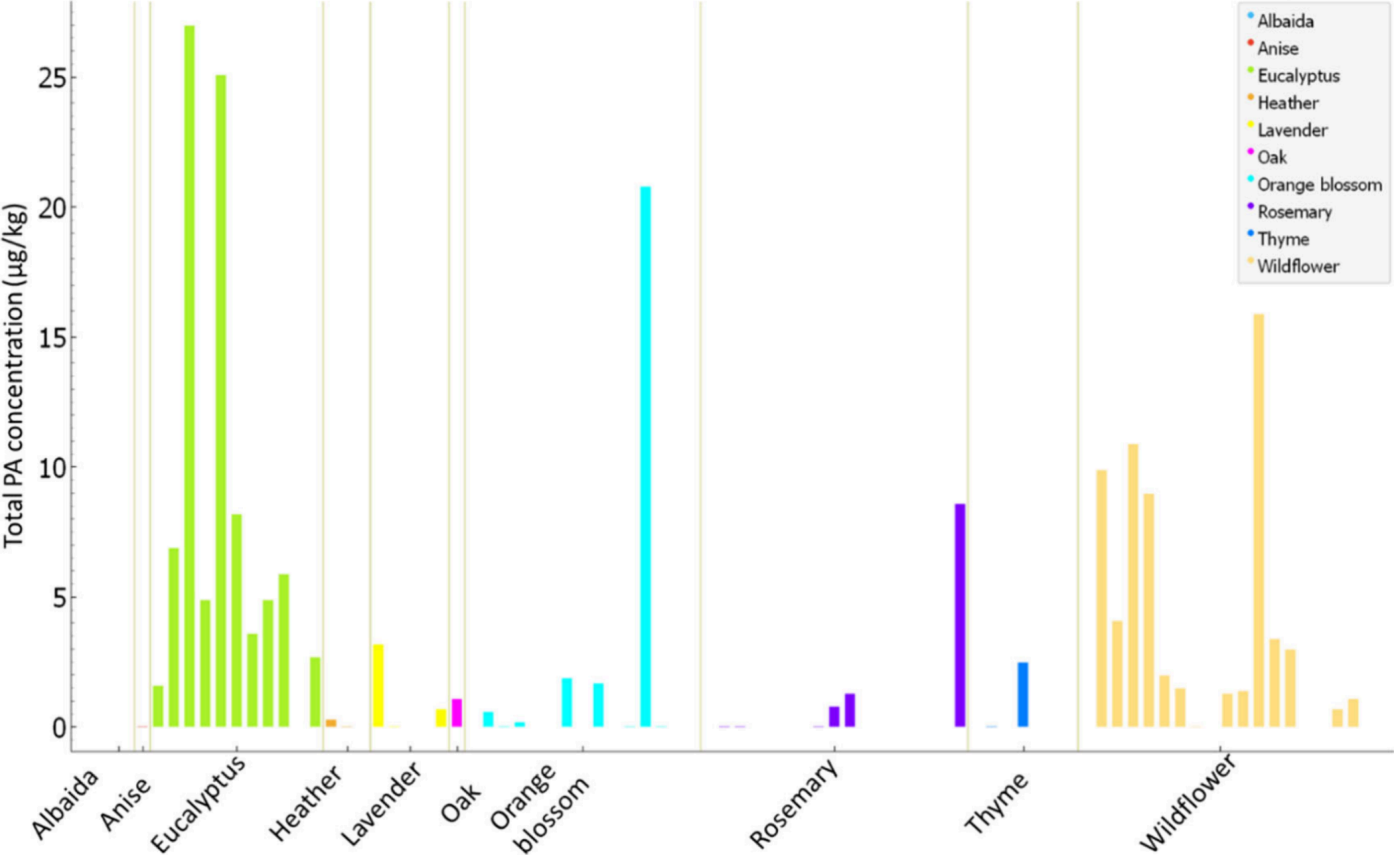
Total PAs concentration (μg/kg) in honey samples, grouped by type of honey. Each bar represents a single sample, and the color coding corresponds to different botanical origins, as indicated in the legend.

Pollen analysis in honey has been employed to identify the plant species that contribute to the presence of PAs in this product. Echimidine and lycopsamine have been linked with plants of the genus , which belongs to the Boraginaceae family, particularly and , respectively.^20,31^ These species are widespread throughout Europe, exposing honeybees and consequently honey of European origin to this plant and its associated PAs.

It is interesting to note that no PANOs were found in the samples above the LOQs, which has also been observed in a previous study that monitored them in honey samples.^29^ In this regard, Kaltner et al.^32^ investigated the stability of PAs and PANOs in relevant food matrices, including honey, while they were stored for a period of 6 months (182 days). A very fast decrease of PANO in honey samples within hours was observed and differences in the degradation rates of a single PANO suggested a compound-dependent derivatization. The levels of PAs did not increase, and thus a simple reduction of PANOs to their corresponding PAs could be excluded. In addition, it is important to note that PAs detected in plants are present as *N*-oxide, whereas they are present mainly as a free base in honey. It is assumed that *N*-oxide reduction may occur in the digestive system of honeybees.^33^

Regarding PA contamination along the honey types, eucalyptus honey showed the highest incidence of PAs. 91% of the eucalyptus honey samples were contaminated with at least 1 PA and presented the highest co-occurrence of PAs with 18% of the positive samples containing between 6 and 10 PAs simultaneously (Figure S4). In addition, a eucalyptus honey sample presented the highest PA contamination, with 27 μg/kg (expressed as the sum of the 35 PAs). Most of the honey samples, regardless of their botanical origin, contained between 1 and 2 PAs, and less frequently between 3 and 5 PAs. Overall, the co-occurrence of the 35 regulated PAs in honey samples is generally low compared to infusions or foods based on herbs.^34^ Our results also suggest that certain types of honey, such as eucalyptus honey, may be more susceptible to PA contamination. However, a larger sample size for each botanical origin is needed to establish a correlation between PA contamination and honey type.

When comparing the incidence of PAs in monofloral and multifloral honey, 53% of the evaluated monofloral honey samples were found to be positive, while 78% of the multifloral honey samples (wildflower) were positive. This distribution was also recently reported by other authors.^35^ This can be explained by the fact that in monofloral production, beekeepers select the predominant flowering periods and the optimal periods to place the hives for bee foraging. In contrast, with multifloral honey, bees can visit a wider variety of plants, increasing their exposure to contamination. However, information on the PAs pattern considering both honey types is still scarce.

According to these results, the presence of PAs/PANOs in honey samples underscores the need for regulatory evaluations and for policymakers to develop and refine the regulatory framework, aligning it with current dietary habits and establishing appropriate maximum limits not only for pollen, but also for honey itself.

#### 3.1.3 Statistical Analysis

An HCA was performed to explore potential groupings among honey samples based on PA profiles. The dendrogram (Figure S2) revealed only partial clustering, with eucalyptus and wildflower honeys forming loosely defined groups, while other types of honey showed substantial overlap. This unsupervised approach highlighted the limited differentiation between honey types, emphasizing the challenges of using these variables for a clear classification. These findings suggested that the PA profiles were not entirely descriptive of honey type, particularly in terms of botanical type.

To further investigate variability in PA profiles, an analysis of variance (ANOVA) was performed between the categorical variable (class, representing honey type) and the numeric variables (analytes). Analytes included were echimidine, echinatine + rinderine, europine, heliosupine, heliotrine, indicine + lycopsamine, intergerrimine, intermedine, lasiocarpine, retrorsine, senecionine, as they were detected as positive, as well as the total concentration. The ANOVA results (Table S5) revealed significant differences in three variables: echinatine + rinderine (*F* = 3.468; *p* = 7.576 × 10^–3^), indicine + lycopsamine (*F* = 7.954; *p* = 6.252 × 10^–6^), and total PA content (*F* = 3.966; *p* = 3.281 × 10^–3^). These results indicated that these specific analytes contributed significantly to the variability among honey types, although their importance was insufficient to establish comprehensive descriptors for honey classification.

Violin plots (Figure 4) were generated to visualize the distributions of the three significant variables identified in the ANOVA across honey types. The distribution of echinatine + rinderine showed a marked enrichment in eucalyptus honey, while indicine + lycopsamine exhibited higher concentrations in both the eucalyptus and wildflower samples. The total PA content revealed that eucalyptus is the honey type with the highest levels, followed by wildflower, and other honey types exhibiting substantially lower concentrations.

**Figure 4:**
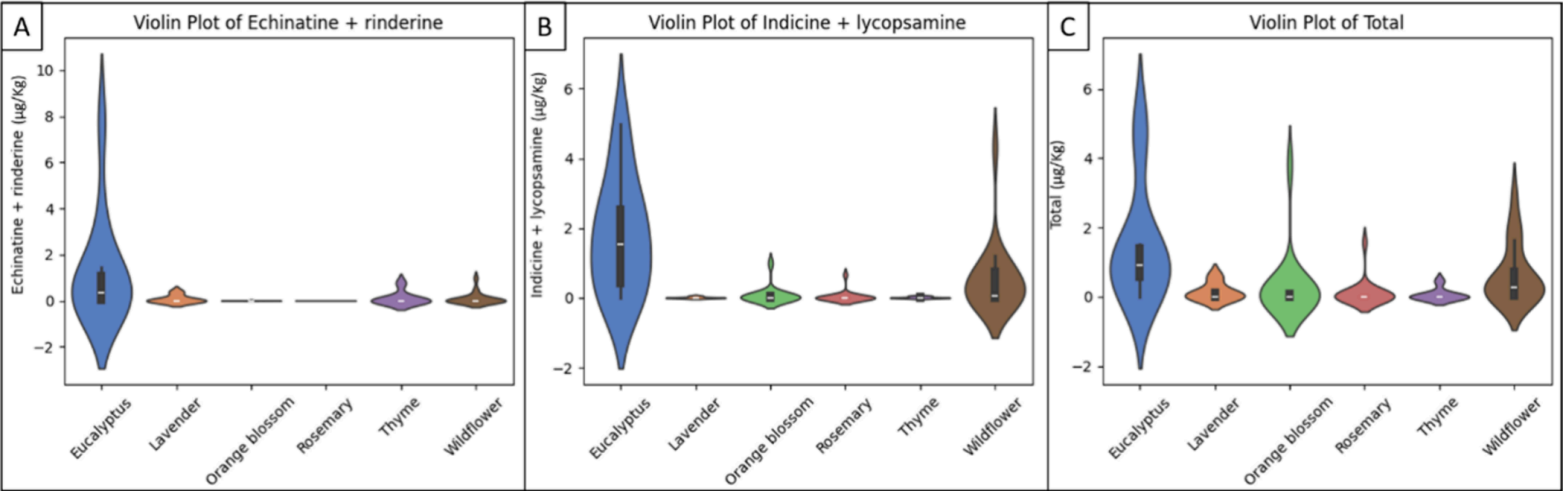
Violin plots showing the distribution of (A) echinatine + rinderine, (B) indicine + lycopsamine, and (C) total PA. Each plot displays the density distribution and variability of PA concentrations within each honey type. The boxes inside the violins represent the interquartile range and median values.

Additionally, a correlation analysis, Pearson correlation coefficient, was performed to examine interrelationships among the analytes. The heatmap visualization (Figure S3) indicated strong positive correlations between certain analytes, such as echinatine + rinderine and intergerrimine, both of which were significantly associated with the total PA content. These correlations suggested that the presence of one analyte was often accompanied by the presence of another analyte with which it was correlated. This raised the possibility that such relationships could be explained chemically, potentially reflecting shared biosynthetic pathways. Echinatine and rinderine are structurally related (isomers), being monoesters and can be produced specially by plants of the genus . In contrast, integerrimine is a cyclic diester primarily produced by plants of the genus . This highlights the diversity of PAs and the variety of PA-producing plants to which bees are exposed.

However, it should be noted that the number of samples analyzed was not large enough to draw definitive conclusions. Despite this limitation, the findings were sufficiently indicative and provided valuable information on the interconnections among analytes in these honey samples.

Finally, supervised classification analyses (such as Random Forest and Support Vector Machine analyses), were tentatively performed to evaluate the potential for predicting honey type based on PA profiles. As expected from the exploratory HCA and the imbalanced data set, these models showed poor performance. While some patterns, such as the enrichment of PAs in Eucalyptus and wildflower honeys, were observed, the results confirmed that PA profiles alone were insufficient to reliably differentiate honey types by botanical type, which aligned with the expected, and more samples are needed to reach further conclusions.

### 3.2 Suspect and Nontarget Screening Analysis

#### 3.2.1 In-House Built Database for Suspect Screening of Honey Samples

In this study, an innovative application of Thermo Fisher’s mzVault software was implemented. Due to the lack of complete reference standards and to explore new strategies leveraging published fragmentation data (e.g., molecular ions, fragments, intensities, adducts, etc.), an alternative approach was implemented. This approach involved generating *in silico* spectra based on bibliographic data. Although these spectra may vary significantly depending on the technique and acquisition conditions, they can still help identify potential common indicators of compounds, similar to how Thermo Fisher’s mzCloud works. An extensive literature search was conducted to identify PAs of interest. Utilizing both molecular ion values and ion ratios, a Python script was developed to generate an *in silico* library. This innovative approach enabled advancement in the identification of compounds in the context of PAs, demonstrating versatility in nonconventional applications.

The creation of the library followed several key steps (Figure S5). First, an initial search was performed in the literature to identify target PAs that had been analyzed using LC–HRMS.^36^ The information gathered was compiled into an Excel table, which included the PA name, molecular formula, molecular ion, fragments, and their relative intensities. In cases where intensity data was not available, all intensities were assigned a value of 100%, allowing for ion detection confirmation even in the absence of intensity similarity. This approach was sufficient for detecting the presence of those ions, although the matching of the ion ratios was challenging due to differences in equipment and parameters.

The Excel table was then processed using the specifically designed Python script (https://github.com/JoseRaulBS/SyntheticMzVault) that converted the data into an MSP file format (essentially a text file, as shown in Figure S5). This MSP file format is compatible with mzVault and was imported to create *in silico* spectra. These *in silico* spectra were then integrated into our workflow in Compound Discoverer, utilizing the mzVault node, allowing seamless incorporation into the overall analytical process.

At the beginning of this process, feature extraction was carried out within the retention time and *m*/*z* ranges of 1–25 min and 70–1000, respectively, for both the ESI+ and ESI– ionization modes. Further extraction parameters included a mass tolerance of 5 ppm, as well as a minimum peak intensity of 1 × 10^5^. A mass tolerance of 5 ppm and a S/N threshold of 1.5 were set for gap filling. The order of preference for assigning the possible identity of the analyte was established as follows: mzVault, mzCloud, Mass List Search, ChemSpider Search, and finally Predicted Composition. This approach prioritized identification based on experimental MS^2^ data using mzVault and mzCloud, followed by common MS1 libraries from Mass List and ChemSpider nodes, and last ensuring that the calculated mass error aligned with the proposed structures derived from the previous methods.

Filters were applied throughout the workflow to reduce the number of candidate analytes, with parameters specifically adapted to the requirements of this study. The signals present in both blank and sample measurements were removed to eliminate background noise. Only features with mass errors between −5 and 5 ppm were retained. A chromatographic peak rating greater than 4 was required, ensuring the retention of real and well-defined signals. The retention time was restricted to the range of 1 to 15 min.

Additionally, a “Class coverage” parameter was incorporated into the workflow to further refine candidate analytes. This parameter involved providing a list of characteristic ions associated with the target analytes, in this case PAs. The logic behind this filter assumes that a valid candidate analyte should contain at least one of these characteristic ions, with more matches indicating a higher likelihood of identification. To implement this, a thorough literature review was conducted to identify commonly reported ions for PAs. A total of 30 characteristic ions, including experimentally observed fragment ions in Table S1, were identified and incorporated into the workflow (Table S6). These ions were utilized to screen and prioritize the resulting analytes, ensuring that only those containing one or more of these ions were retained for further analysis. This approach enhanced the specificity of the workflow by leveraging prior knowledge to filter the candidate pool, aligning well with the study’s objective of identifying PAs with high confidence.

After applying the specified workflow, the number of features was reduced from 4011 to 190. These candidates were carefully reviewed, considering the scores obtained across the libraries. The 35 regulated PAs were identified in the QC and the positive PAs found in the samples by the target approach were confirmed by this strategy, ensuring its reliability. Additionally, several compounds belonging to the pyrrolizidine family, such as retronecine and platynecine, were tentatively identified with level 2 of confidence based on Schymanski levels, which corresponds to a probable structure based on diagnostic MS/MS evidence and spectral similarity.^37^ The in-house library matched 15 PA fragments for the tentatively identified PAs. Since multiple peaks were assigned to these compounds, we decided to obtain their FISh score. The FISh score evaluates the quality of ionization and the strength of detected ion signals, which are critical for obtaining accurate and reliable results in mass spectrometry experiments. In addition, a higher fragmentation coverage means that a greater number of fragment ions are generated and related with the identified molecule, enhancing the accuracy of identification and characterization. In general, a FISH score between 40 and 70% suggest a moderate match, which may support tentative identification when additional evidence For instance, in the case of platynecine, the FISh score was around 40% for several identifications, indicating that several peaks presented a great number of ions that could correspond to fragments of this molecule. The best match of the spectra is shown in Figure 5, where the characteristic fragment ions of the platynecine base (140.1070) were observed.

**Figure 5:**
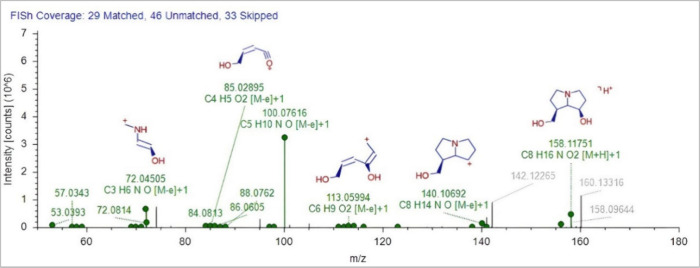
FISh coverage of the putative identified platynecine.

Additionally, other plant toxins, such as tropane alkaloids (TAs), particularly tropinone and atropine, were tentatively identified in the honey samples with a level of confidence 2. These compounds, which are also being investigated alongside PAs, have been previously reported in honey samples at trace levels.^38,39^ The tentative identification of TAs using this strategy further validates the effectiveness of the applied approach, demonstrating its robustness in identifying multiple plant toxins and highlighting the significant interest in their co-occurrence in honey samples.

#### 3.2.2 Nontarget Screening Analysis: Molecular Networking for PA Identification

In this study, a molecular networking strategy was also implemented to investigate the relationships among PAs and related analytes from an unknown–unknown perspective, providing deeper insights into their structural similarities and group affiliations. Using the “Molecular Networks” node in Compound Discoverer, MS/MS spectra and fragmentation patterns were compared through computational algorithms, constructing networks that represented analytes as nodes interconnected based on their spectral similarity.^40,41^ This facilitated the grouping of highly similar compounds, enabling the identification of distinct clusters or families of analytes with shared structural characteristics.

This approach was particularly advantageous given the high number of potential PA candidates and the diversity of compounds detected. By leveraging modern computational technologies, molecular networking efficiently analyzed the extensive MS/MS data, evaluating similarities between analytes in both forward and reverse approaches. It also proved useful for the targeted identification of unknown PAs, even in the absence of common fragment ions or neutral losses, offering a powerful tool to unravel the chemical complexity of the analyzed samples.

To ensure that the scale and potential of molecular networking in this work were not limited by previously applied filters, the class coverage filter was omitted. This adjustment expanded the analyte pool from 785 candidates, creating a comprehensive MS^2^-based fingerprint of the honey matrix. This fingerprint offered a novel perspective on the chemical complexity of the samples. Although this analysis extended beyond the primary scope of this study, clusters were observed that likely represent other families of compounds, suggesting directions for future investigations (Figure 6).

**Figure 6:**
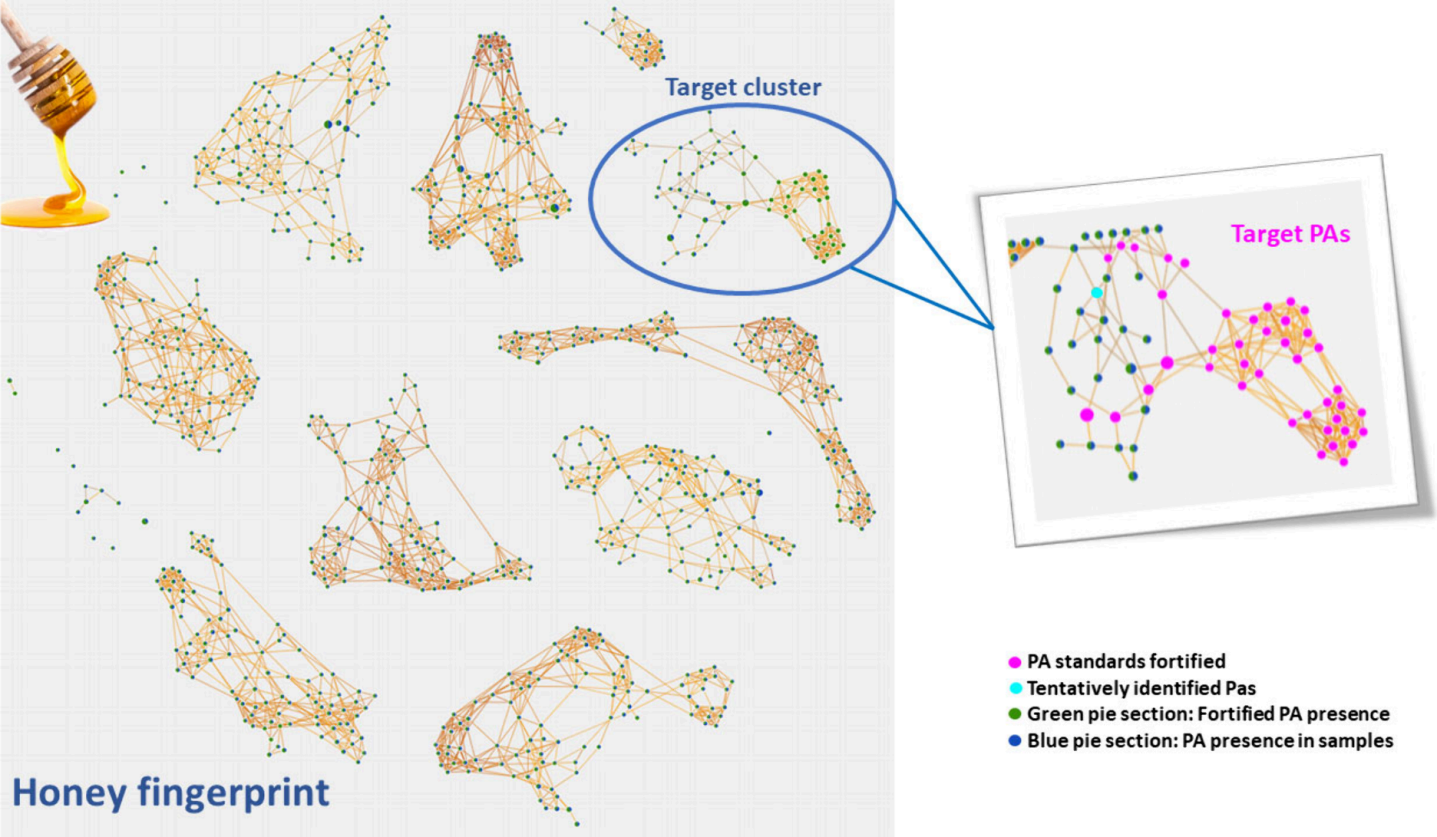
Molecular networking representation of the honey fingerprint, highlighting the target cluster and its integration with the PAs added standards. The network was constructed using a minimum MS^*n*^ similarity score of 20, a minimum MS^*n*^ coverage of 20%, at least 3 matched fragment ions, a maximum of 10 connections per node, and a cluster size limit of 100 nodes.

In this case, optimization of the node connection parameters was carried out to ensure that the analytes intentionally added to the standard sample for targeted analysis were interconnected within the same cluster. This optimization served as a quality control measure, ensuring that the clustering parameters accurately reflected the chemical similarity. The optimized parameters included a minimum MS^*n*^ score of 20, which defined the minimum similarity score to store a connection, ensuring that only connections with sufficient spectral similarity were retained. A minimum MS^*n*^ coverage of 20 was applied, which required that the forward or reverse coverage values met or exceeded this threshold to store a connection. This value was selected after careful optimization to balance sensitivity and specificity, maximizing meaningful clustering while minimizing noise. Additionally, a minimum of 3 matched fragments were required, meaning that connections had to show at least this number of matched fragment ions to be considered valid. These parameters filtered out low-confidence connections, reducing the complexity of the network while retaining meaningful relationships. Node links were limited to a maximum of 10 connections per node, reducing network complexity while retaining meaningful relationships, and a cluster size limit of 100 was imposed to focus on relevant groups of analytes while avoiding overly large and ambiguous clusters. Consequently, analytes interconnected within close proximity were considered potential pyrrolizidine alkaloids by association. From these initial observations, analytes could be tentatively identified using conventional methods, providing a reliable basis for further exploration and characterization.

The network proved useful in narrowing down potential candidates for platynecine (*m*/*z* [M + H]^+^ = 156.1), as only one of the proposed compounds closely aligned with the PA cluster (highlighted in blue in Figure 6). This tentatively identified PA exhibited a retention time (RT = 1.8 min) that differed significantly from the others, probably due to its low polarity and small molecular weight. This finding reinforced confidence in the putative identification of platynecine, especially since it is not typically included and the available literature on it remains limited. To confirm its identity, a commercial standard was acquired and injected into the LC–Orbitrap-MS system, where the matching RT allowed us to assign a level 1 confidence to platynecine. In this case, the PA cluster facilitated the reduction of candidate compounds and provided greater confidence in the identifications made during the suspect screening data analysis. This compound also acts as the basic core (necine) of other PAs belonging to the saturated platynecine type. The lack of the 1,2-double bond in the necine base results in less toxicity, as they are not metabolically activated to hepatotoxic pyrrolic esters.^42^ Identifying the presence of platynecine in honey could provide insights into the types of plants in the surrounding environment that could contribute to PA contamination. However, this secondary metabolite can also be produced by honey bee-associated bacteria.^43^ The presence of bacterial-origin platynecine, with its concentration correlating with mortality, suggests that platynecine may play a role in the direct or indirect biological control of this honey bee ectoparasitic mite, potentially contributing to the reduction of colony collapse disorder (CCD). In any case, its determination in honeybee products is of significant interest.

In the PA cluster, indoline, a benzopyrrole, is directly linked to *N*-nonanoglycine and indirectly to other amino acids. This may be the result of the PA metabolism that leads to the formation of highly reactive compounds, such as pyrrolic esters. These esters can rapidly bind to nucleophilic centers, including DNA, proteins, and amino acids, forming stable pyrrole complexes^44^

This study advances the understanding of PAs in honey through the integration of cutting-edge analytical techniques and comprehensive data analysis. The combination of target, suspect and nontarget screening analysis, including the use of molecular networking with the resulting data, provided a holistic perspective on the occurrence and diversity of these natural toxins in honey samples. The findings revealed a widespread of contamination in certain types of honey, highlighting the role of botanical origins and environmental factors in influencing PA contamination. Statistical analyses highlighted the variability in PA profiles across honey types, but showed limited utility to differentiate botanical origins, which showcased the wide spready of these compounds in such samples.

Given the toxicity and increasing prevalence of PAs in food products, future studies should expand investigations into beehive products and other animal-based foods. This includes increasing the number of samples analyzed and incorporating pollen analysis to better understand contamination sources. In addition, this work reinforces the need for continued research into PA biosynthesis and environmental dynamics, particularly to address gaps in knowledge about their transfer from plants to honeybee products.

The ability to tentatively identify compounds and elucidate structural relationships among them demonstrates the value of applying computational tools alongside high-resolution mass spectrometry, a focus of our ongoing and future research efforts.

## Supplementary Material





## Notes

Retention time (RT) and MS/MS conditions for target PA determination (quantifier transitions in bold) (Table S1), chromatographic separation of 35 target PAs in honey (10 μg/kg) (Figure S1), recoveries at three different concentration levels (*n* = 5) and matrix effect values for each PA (Table S2), precision, expressed as repeatability (intraday; *n* = 5) and intermediate precision (interday; *n* = 9), for each PA (Table S3), hierarchical clustering analysis (HCA) dendrogram showing the grouping of honey samples based on PA profiles (Figure S2), summary of one-way ANOVA results comparing PA concentrations accross different honey samples (Table S5), heatmap of correlation coefficients between analytes measured in honey samples (Figure S3), co-occurrence, expressed in percentage, of PAs in positive honey samples (the number of PAs occurring together indicated in the legend) (Figure S4), workflow for *in silico* library creation for mzVault, with the data collected from the literature compiled in Excel, converted to MSP files using Python, and imported into mzVault for integration into Compound Discoverer (Figure S5), and characteristic ions commonly reported for pyrrolizidine alkaloids in the literature (Table S6) (PDF)
Concentration (μg/kg) of targeted compounds in analyzed honey samples (mean value ± standard deviation from 2 replicates) (Table S4) (XLSX)
