# Supplementary material for: Holistic Approach to Investigate Pyrrolizidine Alkaloids in Honeys from Diverse Botanical Origin: From Target to Suspect and Nontarget Screening Analysis
Source: J Agric Food Chem. 2025 Jun 30;73(32):20410–20. doi: 10.1021/acs.jafc.5c05585 (PMC12356582; doi:10.1021/acs.jafc.5c05585)
Supplement: Supplementary file 1 [file jf5c05585_si_001.pdf]

SUPPORTING INFORMATION

A HOLISTIC APPROACH TO INVESTIGATE PYRROLIZIDINE ALKALOIDS IN HONEYS FROM DIVERSE BOTANICAL ORIGINS: FROM TARGET TO SUSPECT AND NONTARGET SCREENING ANALYSIS

Laura Carbonell-Rozas, José Raúl Belmonte-Sánchez, Paula Soto-Rosiña, Roberto Romero-González, Antonia Garrido Frenich.

Research Group "Analytical Chemistry of Contaminants", Department of Chemistry and Physics, Research Centre for Mediterranean Intensive Agrosystems and Agrifood Biotechnology (CIAIMBITAL), Agrifood Campus of International Excellence (ceiA3), University of Almeria, E-04120 *Almeria,* Spain

#Corresponding Author: lauracr@ual.es

Table S1. Retention time (RT) and MS/MS conditions for target PAs determination (quantifier transitions in bold).

PA	RT (min)	Precursor Ion (*m/z*)	Product Ion (*m/z*)	Fragmentor Voltage (V)	Collision Energy (eV)
Intermedine	3.80	300.2	**94.1**	81	29
			156.0	81	29
			138.0	81	17
Europine	4.00	330.2	**138.1**	148	21
			254.1	148	17
			156.1	148	33
Lycopsamine + indicine	4.06	300.2	**94.1**	70	29
			138.1	70	17
			156.1	70	29
Rinderine + echinatine	4.20	300.2	**138.0**	75	30
			94.1	75	40
			156.0	75	30
Europine N-oxide	4.65	346.2	**172.0**	66	33
			328.1	66	21
			111.1	66	54
Rinderine N-oxide	4.91	316.2	**172.0**	100	40
			94.1	100	50
			111.0	100	50
Echinetine N-oxide	5.05	316.2	**172.1**	164	29
			94.1	164	49
			111.0	164	45
Indicine N-oxide + intermedine N-oxide	5.27	316.2	**172.1**	169	29
			138.1	169	29
			94.1	169	50
Lycopsamine N-oxide	5.76	316.2	**172.1**	174	45
			138.1	174	29
			94.1	174	50
Usaramine	8.38	352.2	**120.1**	190	29
			138.1	190	29
			94.1	190	41
Retrorsine	8.83	352.2	**120.1**	190	33
			94.1	190	41
			67.1	190	50
Usaramine N-oxide	9.10	368.2	**94.1**	184	50
			118.1	184	41
			120.1	184	41
Heliotrine	9.30	314.2	**138.0**	71	17
			156.1	71	29
Retrorsine N-oxide	9.50	368.2	**94.1**	180	50
			118.0	180	37
			120.1	180	41
Spartioidine	9.95	334.2	**120.1**	194	29
			138.1	194	29
			94.1	194	41
Senecifiline	10.37	334.2	**94.1**	194	41
			138.1	194	29
			120.1	194	29

Heliotrine N-oxide	10.95	330.2	**172.1**	150	40
			80.1	150	50
			111.1	150	50
Spartioidine N-oxide	11.57	350.2	**94.1**	185	50
			120.1	185	41
			119.1	185	29
Senecifiline N-oxide	12.12	350.2	**94.1**	180	50
			118.0	180	37
			120.0	180	37
Intergerrimine	14.23	336.2	**120.1**	195	29
			138.1	195	29
			94.1	195	41
Senecivernine	14.81	336.2	**120.0**	190	40
			67.1	190	50
			81.0	190	50
Senecionine	15.45	336.2	**120.0**	180	29
			94.1	180	41
			138.0	180	29
Senecivernine N-oxide	15.95	352.2	**94.1**	200	54
			95.1	200	33
			120.1	200	41
Intergerrimine N-oxide	16.23	352.2	**94.1**	190	50
			118.1	190	37
			120.1	190	41
Senecionine N-oxide	17.10	352.2	**94.1**	75	49
			118.0	75	33
			120.1	75	41
Heliosupine	18.38	398.2	**120.1**	65	29
			336.1	65	17
			220.1	65	17
Senkirkine	18.45	366.2	**168.1**	194	29
			122.1	194	37
			70.1	194	50
Echimidine N-oxide	18.5	414.2	**254.1**	180	33
			396.1	180	25
			83.1	180	50
Echimidine	18.60	398.2	**120.1**	71	25
			55.1	71	49
			83.1	71	29
Heliosupine N-oxide	19.15	414.2	**94.1**	179	50
			119.1	179	41
			138.1	179	33
Lasiocarpine	19.95	412.2	**120.1**	70	29
			336.1	70	17
			220.1	70	17
Lasiocarpine N-oxide	20.45	428.2	**94.1**	195	50
			120.1	195	41
			254.1	195	29

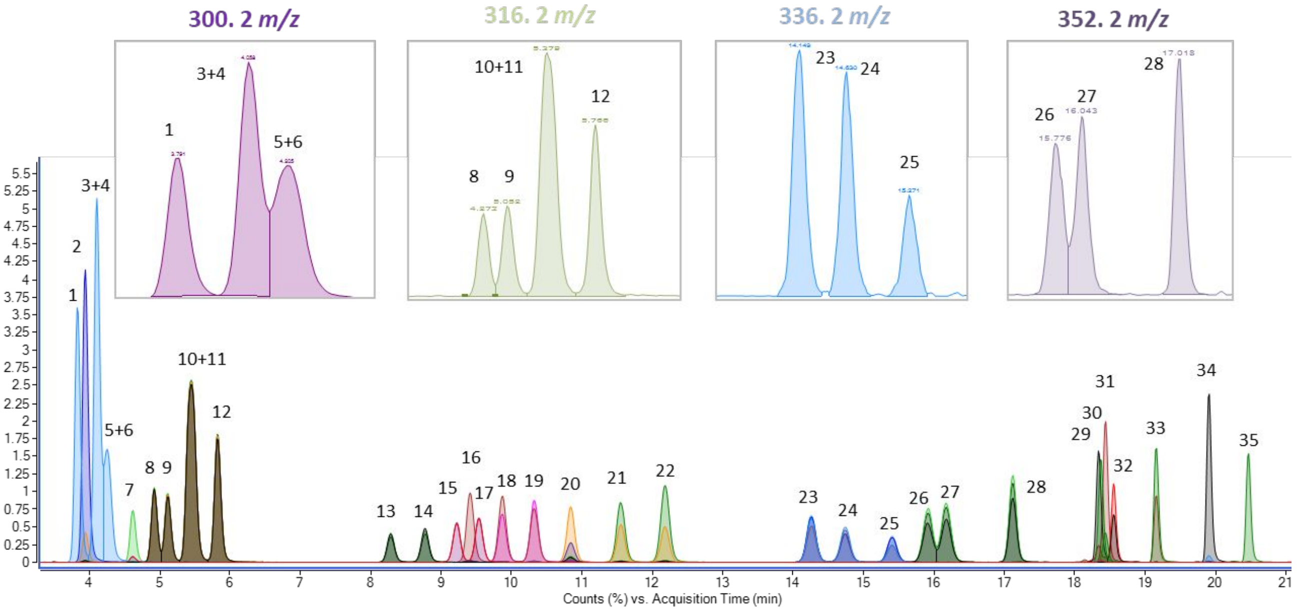

Figure S1. Chromatographic separation of 35 target PAs in honey (10 µg/kg).
Compound code: Intermedine (1); Europine (2); Lycopsamine + Indicine (3,4); Rinderine + Echinatine (5,6); Europine N-oxide (7); Rinderine N-oxide (8); Echinatine N-oxide (9), Intermedine N-oxide + Indicine N-oxide (10,11); Lycopsamine N-oxide (12); Usaramine (13), Retrorsine (14); Usaramine N-oxide (15); Retrorsine N-oxide (16); Heliotrine (17); Spartioidine (18); Seneciphylline (19); Heliotrine N-oxide (20); Spartiodine N-oxide (21); Seneciphylline N-oxide (22); Intergerrimine (23); Senecivernine (24); Senecionine (25); Intergerrimine N-oxide (26), Senecivernine N-oxide (27); Senecionine N-oxide (28); Senkirkine (29); Heliosupine (30); Echimidine N-oxide (31); Echimidine (32); Heliosupine N-oxide (33); Lasiocarpine (34); Lasiocarpine N-oxide (35).

Table S2. Recoveries at three different concentration levels (n=5), and matrix effect values for each PA.

PA	1 µg/kg		10 µg/kg		100 µg/kg		Matrix Effect (%)
	R (%)	RSD (%)	R (%)	RSD (%)	R (%)	RSD (%)	
Echimidine	103.9	6.8	99.4	5.8	102.3	6.6	-2.9
Echimidine N-oxide	86.7	4.0	97.5	4.7	97.8	6.2	3.1
Echinatine + rinderine	97.2	13.8	100.0	3.7	108.9	8.6	-13.6
Echinatine N-oxide	56.9	27.6	81.8	11.0	75.4	9.3	-5.8
Espartioidine	91.6	5.8	100.1	7.3	101.7	13.0	-6.4
Spartioidine N-oxide	86.0	7.8	99.4	7.2	102.1	7.1	0.5
Europine	86.8	7.5	95.0	3.1	98.0	7.5	-10.6
Europine N-oxide	77.0	9.3	86.7	9.3	78.5	3.0	-3.1
Heliosupine	96.0	8.0	99.4	6.5	96.6	10.7	-3.2
Heliosupine N-oxide	99.8	9.7	97.7	7.7	99.0	8.4	0.4
Heliotrine	91.0	7.7	98.9	7.3	100.9	6.0	-5.6
Heliotrine N-oxide	91.9	3.3	98.9	5.8	95.0	11.5	4.4
Indicine + lycopsamine	92.2	8.2	96.7	5.9	98.5	8.6	-8.7
Indicine N-oxide + intermedine N-oxide	83.3	6.2	92.0	8.3	91.2	8.2	-0.9

Intergerrimine	98.3	10.3	99.6	11.8	99.5	10.8	-4.5
Intergerrimine N- oxide	89.0	12.9	100.2	8.4	101.1	4.6	0.7
Intermedine	99.9	5.8	97.0	4.7	95.1	8.8	-9.0
Lasiocarpine	95.9	9.9	98.3	7.7	96.5	13.9	-4.0
Lasiocarpine N-oxide	99.0	1.9	98.7	3.5	103.6	5.5	1.8
Lycopsamine N-oxide	80.6	9.6	87.6	14.1	80.4	9.2	-3.6
Retrorsine	97.3	6.2	97.6	6.1	96.1	7.8	-5.8
Retrorsine N-oxide	89.2	8.7	97.6	7.0	99.2	7.5	-3.6
Rinderine N-oxide	76.7	9.2	83.9	6.7	81.0	5.9	-11.8
Senecionine	79.1	5.0	100.4	4.1	96.1	6.7	-4.4
Senecionine N-oxide	91.1	9.1	100.3	6.1	101.2	7.0	0.5
Senecifiline	87.5	6.4	100.7	7.6	96.2	7.9	-0.9
Senecifiline N-oxide	85.4	10.2	98.8	8.4	97.1	11.9	2.9
Senecivernine	89.3	12.9	98.2	10.0	98.5	10.7	-1.9
Senecivernine N-oxide	93.0	9.3	98.4	4.9	104.8	6.8	-0.8
Senkirkine	93.3	5.2	98.5	5.2	98.9	7.9	-1.2
Usaramine	101.7	9.1	95.3	1.4	100.3	5.0	-6.4
Usaramine N-oxide	76.7	6.5	91.7	11.9	96.2	8.6	-1.5

Table S3. Precision, expressed as repeatability (intra-day, n=5) and intermediate precision (inter-day, n=9) for each PA.

	**Repeatability**			**Intermediate precision**		
**PA**	**1 µg/kg**	**10 µg/kg**	**100 µg/kg**	**1 µg/kg**	**10 µg/kg**	**100 µg/kg**
Echimidine	7.2	3.3	9.4	9.0	8.3	14.0
Echimidine N-oxide	10.0	1.9	6.8	9.2	10.2	14.1
Echinatine + rinderine	16.2	2.9	6.3	12.0	5.1	11.1
Echinatine N-oxide	15.2	7.1	5.8	18.0	9.2	6.0
Espartioidine	3.5	2.3	9.0	5.3	8.6	15.8
Spartioidine N-oxide	5.8	2.4	7.5	7.4	6.6	14.4
Europine	7.8	1.1	8.3	8.8	7.1	14.0
Europine N-oxide	17.9	15.7	17.7	18.1	19.6	17.6
Heliosupine	7.4	2.7	8.3	6.2	7.5	14.6
Heliosupine N-oxide	11.1	4.3	7.7	14.9	8.5	13.9
Heliotrine	8.0	2.2	8.7	6.2	4.7	13.2
Heliotrine N-oxide	3.0	0.9	7.5	3.7	5.5	15.1
Indicine + lycopsamine	8.1	4.4	9.6	7.1	5.1	11.4
Indicine N-oxide + intermedine N-oxide	6.8	4.4	9.7	6.7	6.3	15.7
Intergerrimine	9.5	3.1	10.7	8.7	5.5	14.3
Intergerrimine N- oxide	9.4	2.5	7.1	12.5	6.1	11.0
Intermedine	7.9	1.5	8.3	5.6	5.7	12.7
Lasiocarpine	4.3	5.7	9.6	5.6	7.1	15.8
Lasiocarpine N-oxide	10.1	3.0	8.4	9.6	8.5	14.8

Lycopsamine N-oxide	10.2	1.9	7.4	11.5	9.1	15.9
Retrorsine	14.0	10.5	13.7	12.4	12.5	19.4
Retrorsine N-oxide	7.5	3.4	9.3	13.3	5.2	14.2
Rinderine N-oxide	15.1	3.8	7.8	14.5	9.2	16.0
Senecionine	16.1	19.9	16.6	16.3	20.0	18.2
Senecionine N-oxide	20.3	2.6	11.4	16.2	3.8	14.5
Senecifiline	9.0	1.7	7.5	6.6	6.2	13.0
Senecifiline N-oxide	10.8	2.4	9.4	9.5	7.9	13.5
Senecivernine	5.3	2.5	7.1	9.3	5.3	14.1
Senecivernine N-oxide	18.1	3.4	9.8	12.1	5.1	12.2
Senkirkine	11.8	2.3	8.4	8.8	4.5	13.6
Usaramine	6.6	1.5	7.4	9.0	5.7	13.6
Usaramine N-oxide	10.3	6.8	9.4	10.0	6.3	12.8
Echimidine	12.0	7.3	8.4	9.8	8.6	13.7

Table S4. In excel file: Concentration (μg/kg) of targeted compounds in analyzed honey samples (mean value ± standard deviation from 2 replicates).

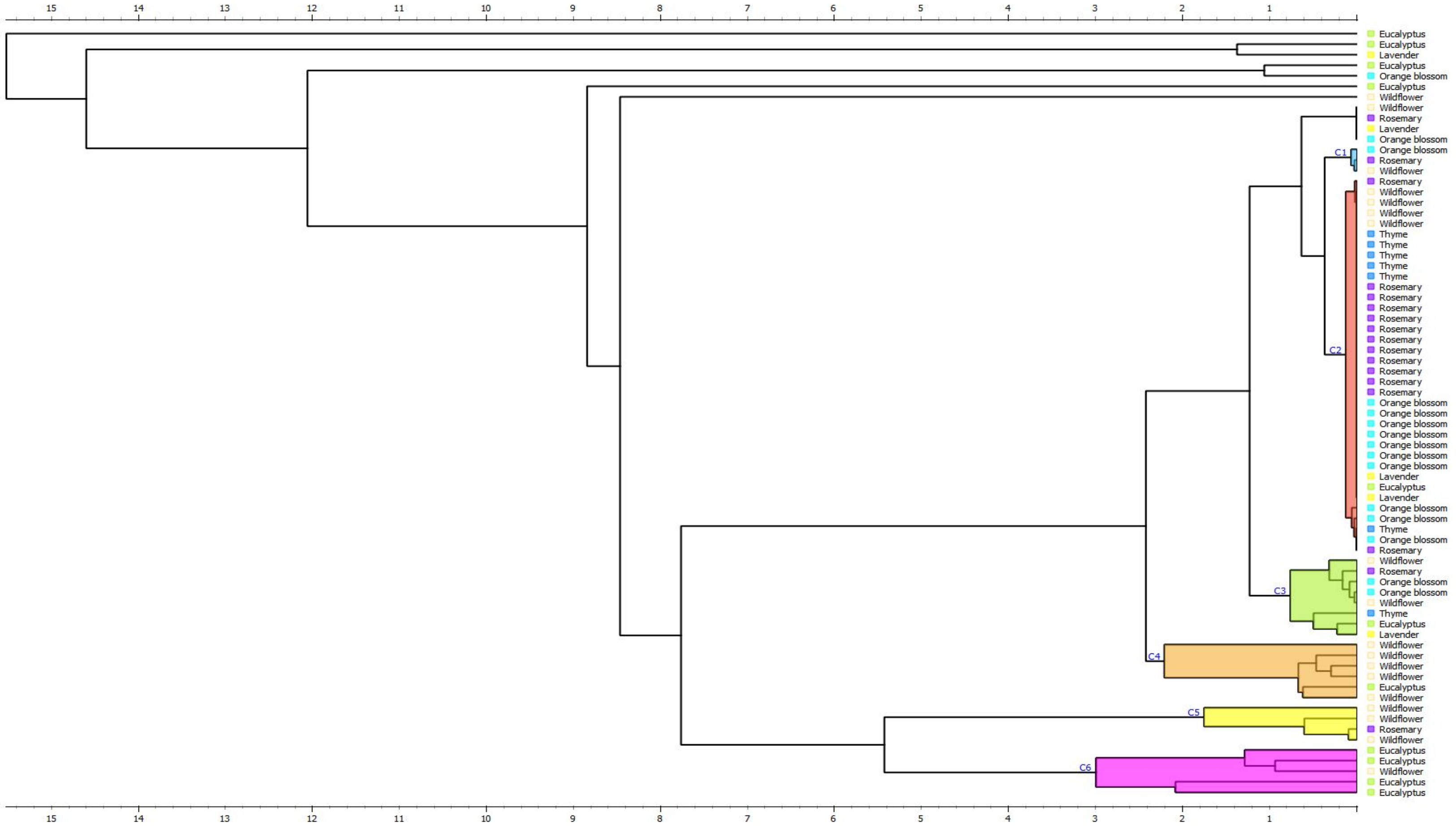

Figure S2. Hierarchical clustering analysis (HCA) dendrogram showing the grouping of honey samples based on PAs profiles

Table S5. Summary of one-way ANOVA results comparing PA concentrations accross different honey samples.

Analyte	F-Statistic (ANOVA)	P-Value
Echimidine	1.011	4.18E-01
Echinatine + rinderine	3.468	7.58E-03
Europine	2.014	8.78E-02
Heliosupine	0.745	5.93E-01
Heliotrine	1.828	1.19E-01
Indicine + lycopsamine	7.954	6.25E-06
Intergerrimine	1.726	1.41E-01
Intermedine	0.924	4.71E-01
Lasiocarpine	1.983	9.24E-02
Retrorsine	1.138	3.49E-01
Senecionine	1.138	3.49E-01
Total	3.966	3.28E-03

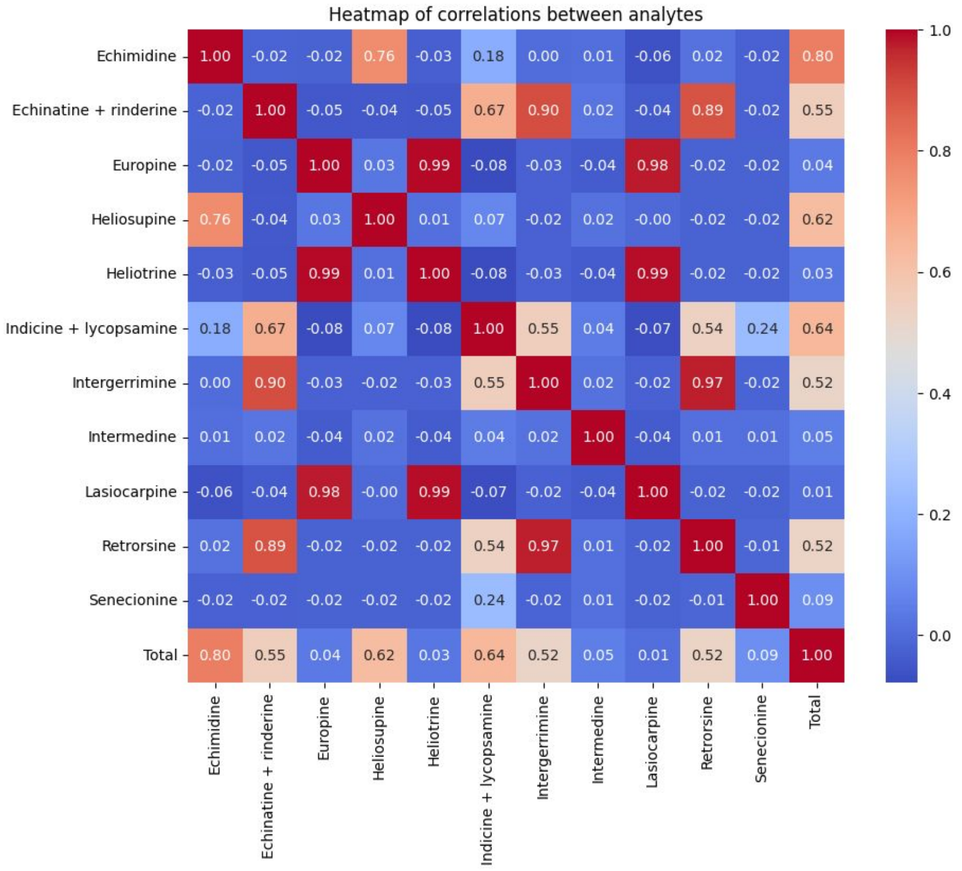

Figure S3. Heatmap of correlation coefficients between analytes measured in honey samples.

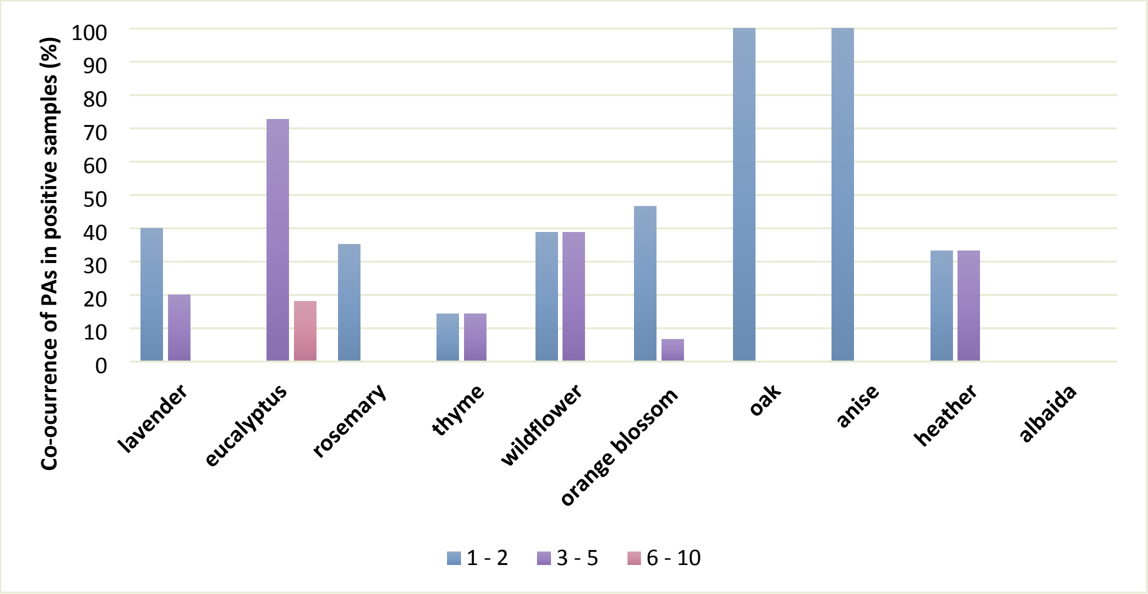

Figure S4. Co-occurrence, expressed in percentage, of PAs in positive honey samples. (The number of PAs occurring together is indicated in the legend).

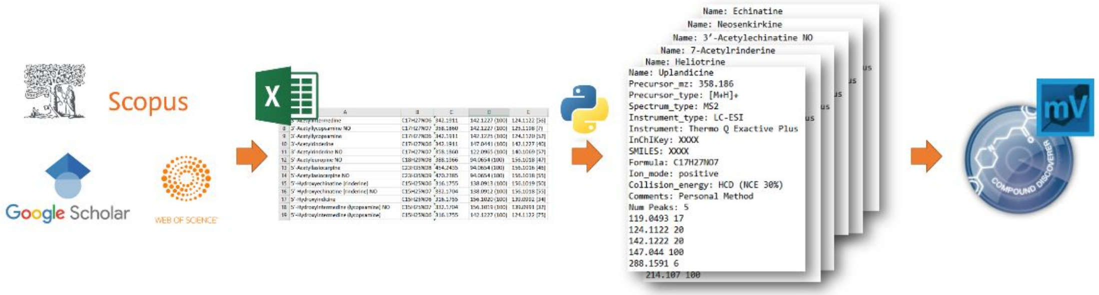

Figure S5. Workflow for in-silico library creation for mzVault. The data collected from the literature was compiled in Excel, converted to MSP files using Python, and imported into mzVault for integration into Compound Discoverer.

Table S6. Characteristic ions commonly reported for pyrrolizidine alkaloids in literature.

PAs common ions (*m/z*)	83.0491; 94.0651; 96.0808; 110.0964; 111.0679; 113.0835; 118.0651; 119.0729; 120.0808; 121.0886; 122.0964; 122.0964; 124.1121; 136.0757; 137.0835; 138.0913; 139.0992; 140.107; 142.1226; 150.0913; 156.1019; 158.1176; 158.1176; 168.1019; 172.0968; 174.1125; 180.1019; 198.1125; 214.1074; 220.1332; 238.1438; 254.1387
